# Once‐Weekly Insulin Versus Once‐Daily Insulin for Type 1 Diabetes Treatment: A Systematic Review and Meta‐Analysis of Randomised Controlled Trials

**DOI:** 10.1002/edm2.70048

**Published:** 2025-04-04

**Authors:** Obieda Altobaishat, Ahmed Farid Gadelmawla, Suliman Almohtasib, Husam Abu Suilik, AlMothana Manasrah, Mohamed Abouzid, Mustafa Turkmani, Mohamed Abuelazm

**Affiliations:** ^1^ Faculty of Medicine Jordan University of Science and Technology Irbid Jordan; ^2^ Faculty of Medicine Menoufia University Menoufia Egypt; ^3^ Faculty of Medicine University of Jordan Amman Jordan; ^4^ Faculty of Medicine The Hashemite University Zarqa Jordan; ^5^ Internal Medicine Department United Health Services–Wilson Medical Center Johnson City New York USA; ^6^ Department of Physical Pharmacy and Pharmacokinetics, Faculty of Pharmacy Poznan University of Medical Sciences Poznan Poland; ^7^ Doctoral School Poznan University of Medical Sciences Poznan Poland; ^8^ Faculty of Medicine Michigan State University East Lansing Michigan USA; ^9^ Department of Internal Medicine McLaren Health Care Oakland Michigan USA; ^10^ Faculty of Medicine Tanta University Tanta Egypt

**Keywords:** diabetes mellitus, insulin, meta‐analysis, T1DM

## Abstract

**Background:**

Type 1 diabetes mellitus (T1DM) represents a considerable global health burden, affecting approximately 5%–10% of individuals with diabetes. Once‐weekly basal insulin could substantially reduce the number of injections for T1DM patients from 365 daily to 52 weekly doses annually. Therefore, this meta‐analysis compares the safety and efficacy of once‐weekly insulin formulations.

**Methods:**

The systematic review and meta‐analysis included the relevant randomised controlled trials (RCTs) retrieved from PubMed, EMBASE, Web of Science, Cochrane, and SCOPUS databases until September 2024. The meta‐analysis was performed using (RevMan 5.4.1). The study protocol was registered on PROSPERO (CRD42024603022).

**Results:**

Three RCTs comprising 1724 participants were included. Once‐daily insulin significantly decreased glycated haemoglobin (HbA1c) compared to once‐weekly insulin (estimated treatment difference: 0.09%, 95% CI [0.07, 0.11], *p* < 0.00001). Fasting blood glucose levels were comparable between the once‐weekly and once‐daily insulin groups (estimated treatment difference: 0.44 mg/dL, 95% CI [−0.64, 1.52], *p* = 0.42).

Once‐weekly insulin was associated with a significant increase in the incidence of injection site reactions (RR: 3.48 with 95% CI [1.30, 9.31], *p* = 0.01), serious adverse events (RR: 1.55 with 95% CI [1.09, 2.19], *p* = 0.01), and treatment‐emergent adverse events (RR: 1.12 with 95% CI [1.02, 1.23], *p* = 0.02), while no significant difference was observed in hypersensitivity reactions (RR: 1.04 with 95% CI [0.78, 1.38], *p* = 0.79).

**Conclusion:**

Once‐daily insulin has demonstrated slightly superior HbA1c reduction, while once‐weekly insulin offers potential advantages in patient adherence. However, these benefits must be weighed against an increased risk of injection site reactions and nocturnal hypoglycemia. Although once‐weekly insulin is more convenient, treatment decisions should consider individual patient factors such as hypoglycemia risk and tolerance to injection reactions.

## Graphical abstract

Once‐daily insulin provides superior HbA1c reduction compared to once‐weekly insulin in type 1 diabetes, while fasting blood glucose levels remain comparable. Although once‐weekly insulin may improve adherence, it is associated with higher rates of injection site reactions and adverse events, requiring careful consideration of patient‐specific factors in treatment decisions.

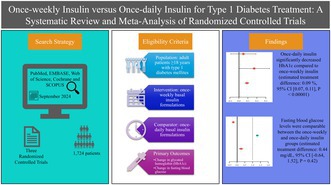

## 1 Introduction

Type 1 diabetes mellitus (T1DM), though less common than type 2 diabetes, affects approximately 5%–10% of individuals with diabetes and is characterised by absolute insulin deficiency due to autoimmune destruction of pancreatic islet β‐cells. This leads to impaired glucose metabolism and necessitates lifelong insulin therapy [1]. Despite advancements such as continuous glucose monitoring (CGM), maintaining optimal glycemic control remains challenging due to complex intra‐daily and day‐to‐day glucose fluctuations influenced by factors like exercise, food intake, stress and hormonal changes [2, 3]. Traditional reliance on glycated haemoglobin (HbA1c) as the primary marker of glucose control may not fully capture these fluctuations, which contribute to complications in T1DM [4]. Addressing glycemic variability is crucial for improving patient outcomes and achieving more stable glucose control [5].

Real‐world evidence suggests that reducing the frequency of insulin administrations, such as once‐weekly insulin formulations, can significantly improve treatment adherence and, consequently, better glycemic control. This is especially important in the management of T1DM, where tight glycemic control is required to avoid complications like diabetic ketoacidosis (DKA) [5, 6]. Recent advancements in long‐acting insulin formulations have focused on extending insulin activity while ensuring a stable pharmacokinetic and pharmacodynamic profile. Innovations such as insulin icodec and insulin efsitora alfa incorporate structural modifications, including albumin binding and Fc fusion technology, to prolong the half‐life and provide sustained glucose‐lowering effects. These developments aim to reduce injection frequency, enhance adherence, and minimise glycemic variability.

Additionally, insulin receptor affinity and depot stability improvements have contributed to more predictable glucose control, addressing key challenges in T1DM management [7, 8, 9]. Studies have shown that it offers non‐inferior glycemic control compared to daily insulin degludec. Introducing once‐weekly basal insulin could significantly reduce the number of injections for T1DM patients from 365 daily doses to only 52 weekly doses annually [7].

Our study aims to address this significant advancement by performing a meta‐analysis to compare the efficacy of once‐weekly insulin formulations, such as icodec and BIF, with conventional once‐daily insulin options, including degludec. The findings from this analysis will provide critical insights to support clinical decision‐making, inform the development of treatment guidelines, and influence healthcare policy, ultimately aiming to enhance personalised and effective care for individuals living with T1DM.

## 2 Methodology

### 2.1 Protocol Registration

Our review was conducted following the Cochrane Handbook for Systematic Reviews and Meta‐Analyses [10] and reported following the Preferred Reporting Items for Systematic Reviews and Meta‐Analyses (PRISMA) guideline [11]. We published the protocol on PROSPERO for this meta‐analysis and pre‐registered it (CRD42024603022).

### 2.2 Data Sources and Search Strategy

Through September 12th, 2024, O.A. and A.M. performed an electronic search across five databases: PubMed (MEDLINE), Scopus, Web of Science (WoS), Cochrane Central Register of Controlled Trials (CENTRAL), and Excerpta Medica database (EMBASE). For every database, we changed the search parameters and keywords. The searchs outcomes are shown in (Table S1).

### 2.3 Eligibility Criteria and Study Selection

We included studies that met the following PICOS criteria: Population (adult patients ≥ 18 years with T1DM), Intervention (once‐weekly basal insulin formulations), and Comparison (once‐daily basal insulin formulations). Primary outcomes: (change in HbA1c and change in fasting blood glucose after an overnight fast from baseline to end of the study period), and secondary outcomes: change in body weight, treatment‐emergent adverse events, serious adverse events, death, injection site reaction, hypersensitivity reaction, hypoglycemia level 1 a glucose alert value of 54–70 mg/dL (3.0–3.9 mmol/L), hypoglycemia level 2 a glucose level of < 54 mg/dL (< 3.0 mmol/L), hypoglycemia level 3 a severe event characterised by altered mental or physical status requiring external assistance for recovery, regardless of glucose measurement, combined level 2 or 3 hypoglycemia, nocturnal hypoglycemia level 1, nocturnal hypoglycemia level 2, nocturnal hypoglycemia level 3, combined level 2 or 3 nocturnal hypoglycemia, definitions for nocturnal hypoglycemia (Levels 1, 2, and 3) are identical to those for hypoglycemia but specifically occur during sleep (between bedtime and waking). Only RCTs with a minimum duration of 12 weeks were included to ensure adequate assessment of glycaemic control and safety outcomes.

The following criteria were used to filter out studies: (1) non‐original studies (e.g., book chapters, reviews, correspondence, letters to editors, commentaries, press articles, guidelines, etc.,); (2) study designs other than RCTs; (3) studies involving duplicate or overlapping datasets; (4) studies with fewer than 10 participants; (5) in vitro experiments and non‐human studies; and (6) studies not reported in English.

### 2.4 Study Selection

The review procedure was carried out using the Covidence online tool. After eliminating duplicates, the authors (H.A. and A.M.) reviewed the retrieved records separately. The entire texts of the documents that initially satisfied the eligibility standards were reviewed by (H.A. and A.M.) during the full‐text screening. Every argument was settled by consensus and discussion with O.A.

### 2.5 Data Extraction

Following the retrieval of all the texts of the relevant studies, we carried out a pilot extraction to arrange the data extraction sheet appropriately. There are three primary sections to the Excel (Microsoft, USA) data extraction sheet. The summary features of the included studies were presented in the first section (name of the first author, year of publication, country, name of the journal, study design, eligible HbA1c range, target fasting glucose, bolus dose of basal insulin, previous basal insulin therapy, use of short‐acting bolus insulin, insulin dose‐adjustments, follow up duration, primary outcome). The second part included the baseline information of the participants (sample size, age, gender, ethnicity, race, duration of diabetes, body mass index (BMI), baseline HbA1c, baseline fasting glucose, carb counting, CGM, and basal insulin). Finally, the third part included outcomes data as previously described. (H.A. and A.M.) completed the data extraction process. A senior author (O.A.) was consulted, and agreements were established to settle disputes.

### 2.6 Risk of Bias

Using the Cochrane RoB2 tool, (H.A. and A.M.) independently assessed the studies' quality [12]. They evaluated the following five domains: the risk of bias associated with the randomisation process, deviation from the intended intervention, missing outcome data, measuring the outcome, and choosing the reported results. The first author (O.A.) was consulted, and agreements were established to settle disputes.

### 2.7 Statistical Analysis

We utilised Review Manager (RevMan) software 5.4.1 [13]. Outcome measures were assessed using the risk ratio (RR) and its corresponding 95% confidence interval (CI). To account for variations in study designs and participant characteristics, we employed a random‐effects model to address potential heterogeneity. Heterogeneity was evaluated using the *I* ^2^ statistic, which quantifies the percentage of the total variability in effect estimates across studies attributed to heterogeneity rather than chance. We considered a significance level of *p* < 0.1 for the *I* ^2^ statistic to indicate notable heterogeneity. In instances of significant heterogeneity, we conducted a leave‐one‐out meta‐analysis. The DerSimonian and Laird method was used to estimate the between‐study variance (tau‐squared) within the random‐effects model. Statistical significance for the overall effect size was determined by a *p*‐value below 0.05.

## 3 Results

### 3.1 Search Results and Study Selection

**FIGURE 1:**
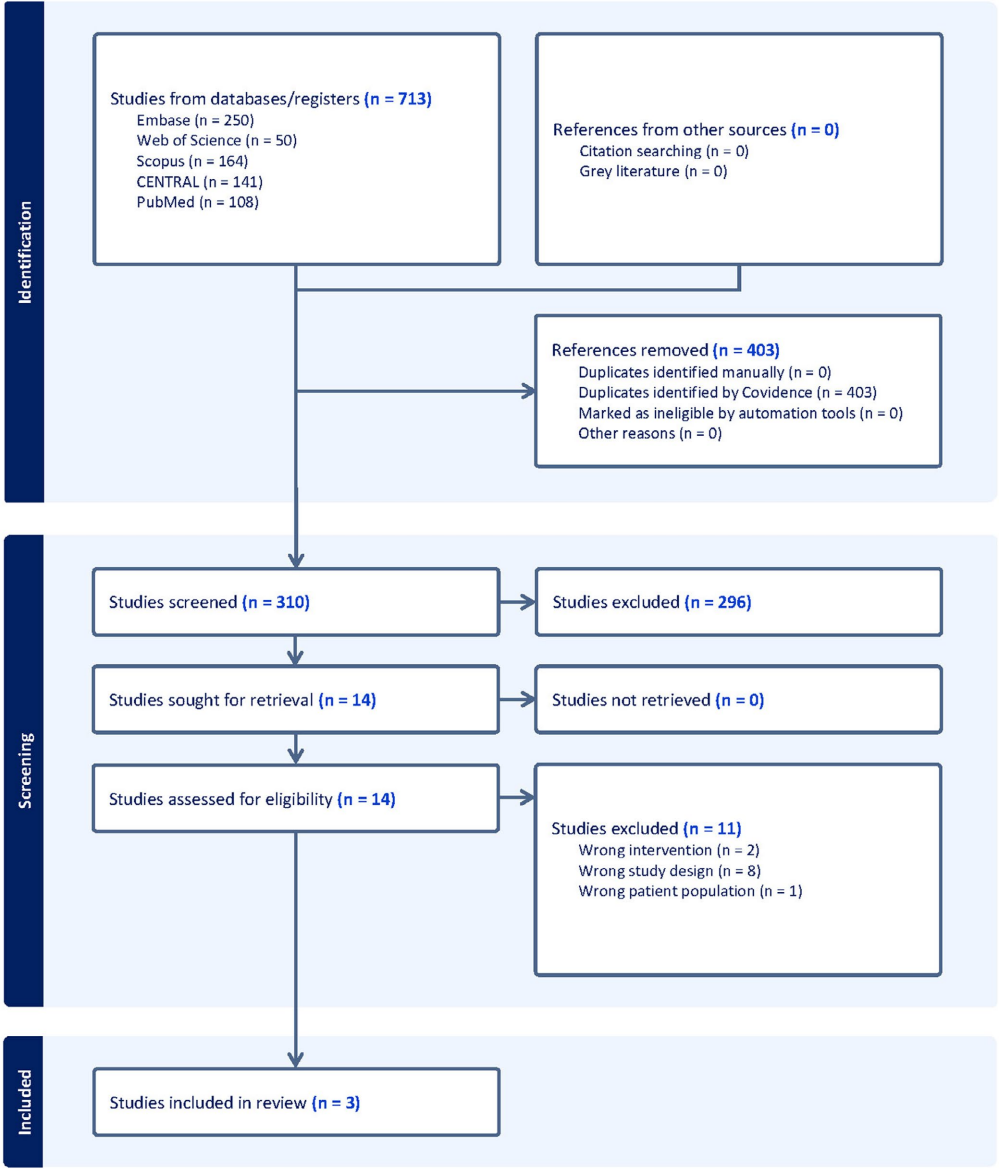
PRISMA flow chart of the screening process.

Our search retrieved 713 studies after searching the databases. After removing duplicates, there were 310 studies. Following title and abstract screening, 14 studies were evaluated in full text. Of these, three studies [7, 14, 15] were included in this systematic review and meta‐analysis. The PRISMA flow diagram is shown in (Figure 1).

### 3.2 Characteristics of Included Studies

A total of three RCTs with 1724 participants were included. Two were phase III RCTs, while Kazda et al. was a phase II trial. The follow‐up duration ranged from 26 weeks to 57 weeks. In the once‐weekly group, two studies used insulin alpha efsitora, while one used insulin icodec combined with aspart in the intervention group. In the once‐daily group, two studies used degludec, while Jones et al. used aspart in combination with degludec in the comparator group. The mean age of the participants ranged from 44.1 to 47.4 years, and the BMI ranged from 25.9 to 27.5 kg/m^2^. Further details of the included studies' summary and the participants' baseline characteristics are demonstrated in Tables 1 and 2. Moreover, key findings (e.g., HbA1c changes, hypoglycemia rates, and adverse effects) across the studies analysed are shown in Table 3.

**TABLE 1:** Summary characteristics of the included RCTs.

Study ID	Study design	Country	No. of participants	Intervention	Control	Eligible HbA1c range	Target fasting glucose	Bolus dose of basal insulin	Previous basal insulin therapy	Use of short‐acting bolus insulin	Follow‐up duration	Primary outcome	Overall ROB
Jones et al. (2023)^a^ [15]	Phase IIIa RCT	Austria, Canada, Germany, India, Italy, Japan, the Netherlands, Russia, Spain, Turkey, the UK, and the USA	582	Once‐weekly icodec in combination with insulin aspart (two or more daily injections)	Once‐daily degludec in combination with insulin aspart (two or more daily injections)	< 10.0%	4.4–7.2 mmol/L (80–130 mg/dL)	Yes	Yes	Yes	57 weeks	HbA1c change from baseline to week 26	Performance bias, High
Kazda et al. (2023) [7]	Phase II RCT	Spain, Austria, Germany, Puerto Rico, and the USA	249	BIF once weekly	Degludec once daily	5.6%–9.5%	80–100 mg/dL	No	Yes	Yes	26 weeks	HbA1c change from baseline to week 26	Performance bias, High
Bergenstal et al. (2024) [14]	Phase III RCT	Argentina, Japan, Poland, Slovakia, Taiwan, and the USA	893	Once‐weekly efsitora	Once‐daily degludec	7.0%–10.0%	80–120 mg/dL	Yes	Yes	Yes	52 weeks	HbA1c change from baseline to week 26	Performance bias, High

Abbreviations: BIF, basal insulin Fc; HbA1c, haemoglobin A1c; RCT, randomised controlled trial; ROB, risk of bias.

^a^ Insulin dose adjustment was only reported in this study. Participants were switched to degludec to achieve a pre‐breakfast self‐measured blood glucose value of 4–7 mmol/L. The starting dose for the first injection was calculated as the pre‐trial daily basal insulin dose multiplied by seven, with a one‐time additional dose of 50% for participants with a screening HbA1c of < 8.0% and 100% for those with a screening HbA1c of ≥ 8.0%.

**TABLE 2:** Baseline characteristics of the included RCTs.

Study ID	Groups	No. of participants	Age in years mean (SD)	Male *n* (%)	Ethnicity *n* (%)			Race *n* (%)			Diabetes duration mean (SD)	BMI mean (SD)	Baseline HbA1c, % mean (SD)	Baseline fasting glucose mg/dL mean (SD)	Carb counting *n* (%)	Continuous glucose monitoring *n* (%)	Basal insulin			
					Hispanic or Latino	Non‐Hispanic or Latino	Black or African American	White	Asian	Others							Glargine *n* (%)	Detemir *n* (%)	Degludec *n* (%)	Other *n* (%)
Jones et al. (2023) [15]	Insulin icodec	290	44.1 (14.1)	165 (57)	NA	NA	9 (3)	230 (79)	51 (18)	NA	20.0 (13.2)	26.8 (5.0)	7.59 (0.96)	179 (74)	131 (45)	NA	97 (33)	20 (7)	120 (41)	3 (1)
	Insulin degludec	292	44.3 (14.1)	172 (59)	NA	NA	2 (1%)	218 (75)	72 (25)	NA	19.0 (12.9)	26.2 (4.5)	7.63 (0.93)	172 (72)	134 (46)	NA	91 (31)	29 (10)	113 (39)	2 (1)
Kazda et al. (2023) [7]	Insulin degludec	126	47.4 (13.7)	78 (61.9)	10 (7.9)	116 (92.1)	NA	NA	NA	NA	22.3 (13.9)	27.2 (4.1)	7.5 (0.9)	159.3 (67.1)	NA	NA	64 (50.8)	4 (3.2)	58 (46.0)	NA
	Efsitora	123	44.4 (14.9)	74 (60.2)	22 (17.9)	100 (81.3)	NA	NA	NA	NA	21.7 (13.3)	27.5 (4.0)	7.5 (0.9)	165.5 (68.4)	NA	NA	66 (53.7)	5 (4.1)	52 (42.3)	NA
Bergenstal et al. (2024) [14]	Efsitora	343	44.4 (14.2)	193 (56)	121 (35)	221 (64)	11 (3)	259 (76)	69 (20)	NA	18 (12.5)	26.5 (4.0)	7.88 (0.75)	157.2 (68.5)	160 (47)	118 (34)	168 (49)	12 (3)	163 (48)	NA
	Insulin degludec	349	43.6 (14.0)	191 (55)	116 (33)	230 (66)	13 (4)	262 (75)	73 (21)	NA	18.6 (11.8)	25.9 (4.1)	7.94 (0.72)	164 (71.5)	165 (47)	123 (35)	175 (50)	17 (5)	157 (45)	NA

Abbreviations: BMI, body mass index; HbA1c, haemoglobin A1c; SD, standard deviation.

**TABLE 3:** Summary of the key findings across the studies analysed.

Study ID	Groups	Sample size	Follow‐up duration in weeks	HbA1c change (%) ETD	Fasting blood glucose change ETD	Hypoglycemia Rates %	Nocturnal hypoglycemia %	Serious adverse effects %
Jones et al. (2023) [15]	Insulin icodec	290	57	−0.37	0.58	Level 1: 99.31% Level 2: 90.34% Level 3: 4.48%	Level 1: 81.72% Level 2: 58.97% Level 3: 1.38%	8.28%
	Insulin degludec	292		−0.54	−1.88	Level 1: 98.97% Level 2: 85.62% Level 3: 4.11%	Level 1: 72.26% Level 2: 47.95% Level 3: 1.37%	6.85%
Kazda et al. (2023) [7]	Insulin degludec	126	26	−0.04	−0.41	Level 1: 100% Level 2: 93.10%	Level 1: 97.41 Level 2: 72.41%	3.60%
	Efsitora	123		−0.13	−0.67	Level 1: 100% Level 2: 86.18	Level 1: 93.5% Level 2: 56.91%	3.17%
Bergenstal et al. (2024) [14]	Efsitora	343	52	−0.39	−1.47	Level 1: 99.13% Level 2: 78.72% Level 3: 10.20%	Level 1: 76.38%	13.12%
	Insulin degludec	349		0.9	−1.23	Level 1: 96.56% Level 2: 84.53% Level 3: 3.15%	Level 1: 70.49%	6.88%

Abbreviation: ETD, estimated treatment difference.

### 3.3 Risk of Bias

**FIGURE 2:**
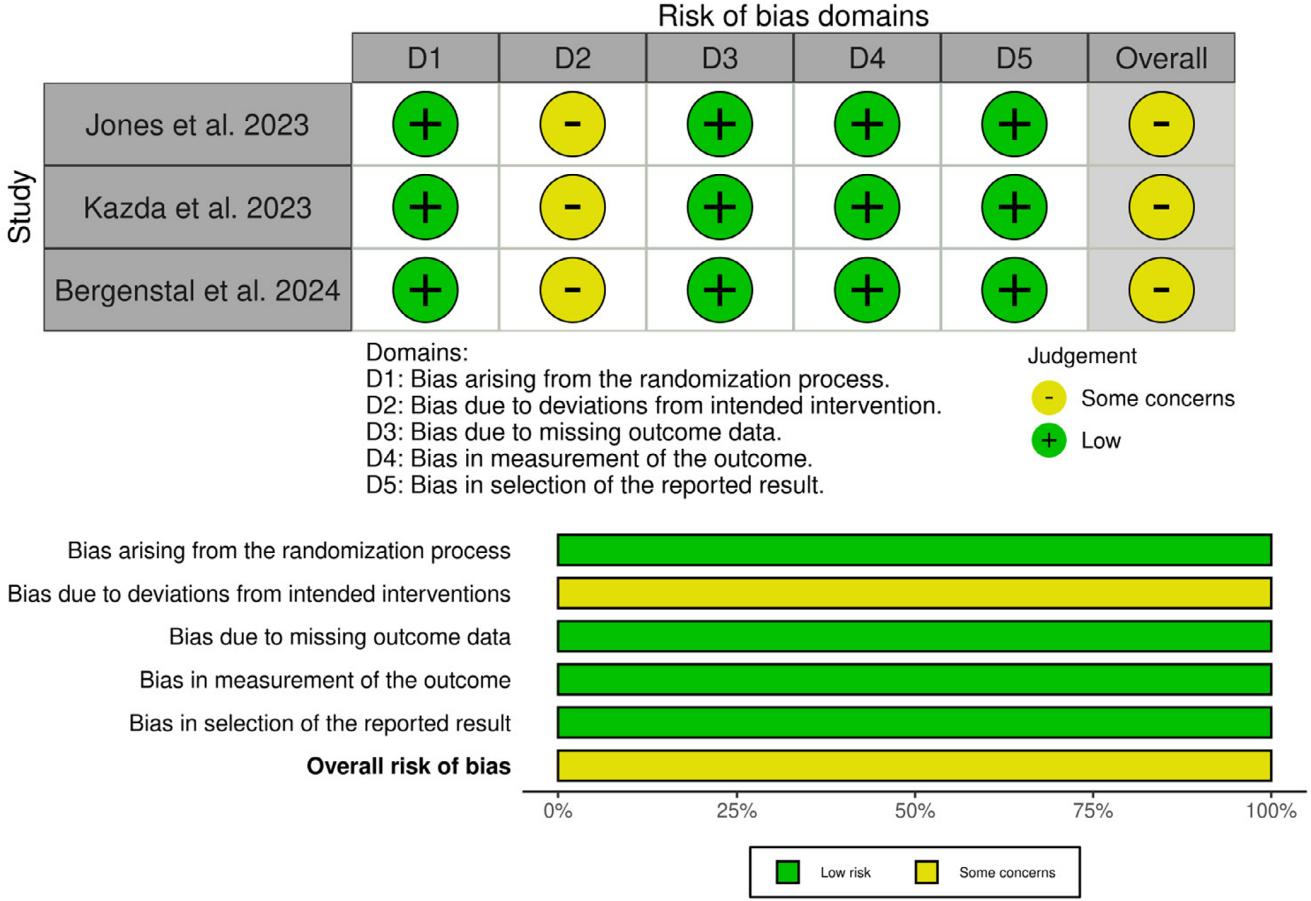
Quality assessment of risk of bias in the included trials. A schematic representation of risks (low = green, unclear = yellow, and high = red) for specific types of biases in each of the studies in the review.

All three studies showed some concerns, mainly due to the unclear risk of deviations from the intended intervention, primarily due to the open‐label study design, which may have influenced participant and investigator behaviour (Figure 2).

### 3.4 Primary Outcomes

**FIGURE 3:**
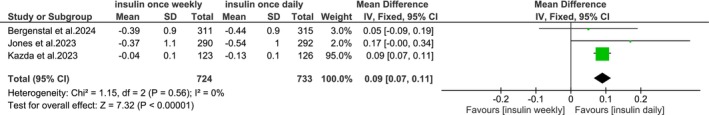
Forest plot of the differences in HbA1c levels.

The pooled data from the three studies, including 1457 participants, showed that once‐daily insulin significantly decreased HbA1c compared to once‐weekly insulin (estimated treatment difference (ETD): 0.09% with 95% CI [0.07, 0.11], *p* < 0.00001) (Figure 3). The pooled studies were homogenous (*p* = 0.56, *I* ^2^ = 0%).

### 3.5 Secondary Efficacy Outcomes

**FIGURE 4:**
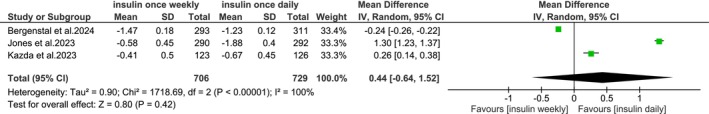
Forest plot of the differences in fasting blood glucose levels.

The pooled data from the three studies, including 1435 participants, showed that once‐weekly insulin was associated with no statistically significant difference in the fasting blood glucose levels compared to once‐daily insulin (ETD: 0.44 mg/dL with 95% CI [−0.64, 1.52], *p* = 0.42) (Figure 4). The pooled studies were heterogeneous (*p* < 0.00001, *I* ^2^ = 100%), and the heterogeneity could not be solved through the leave‐one‐out sensitivity analysis (Table S2).

### 3.6 Secondary Safety Outcomes

**FIGURE 5:**
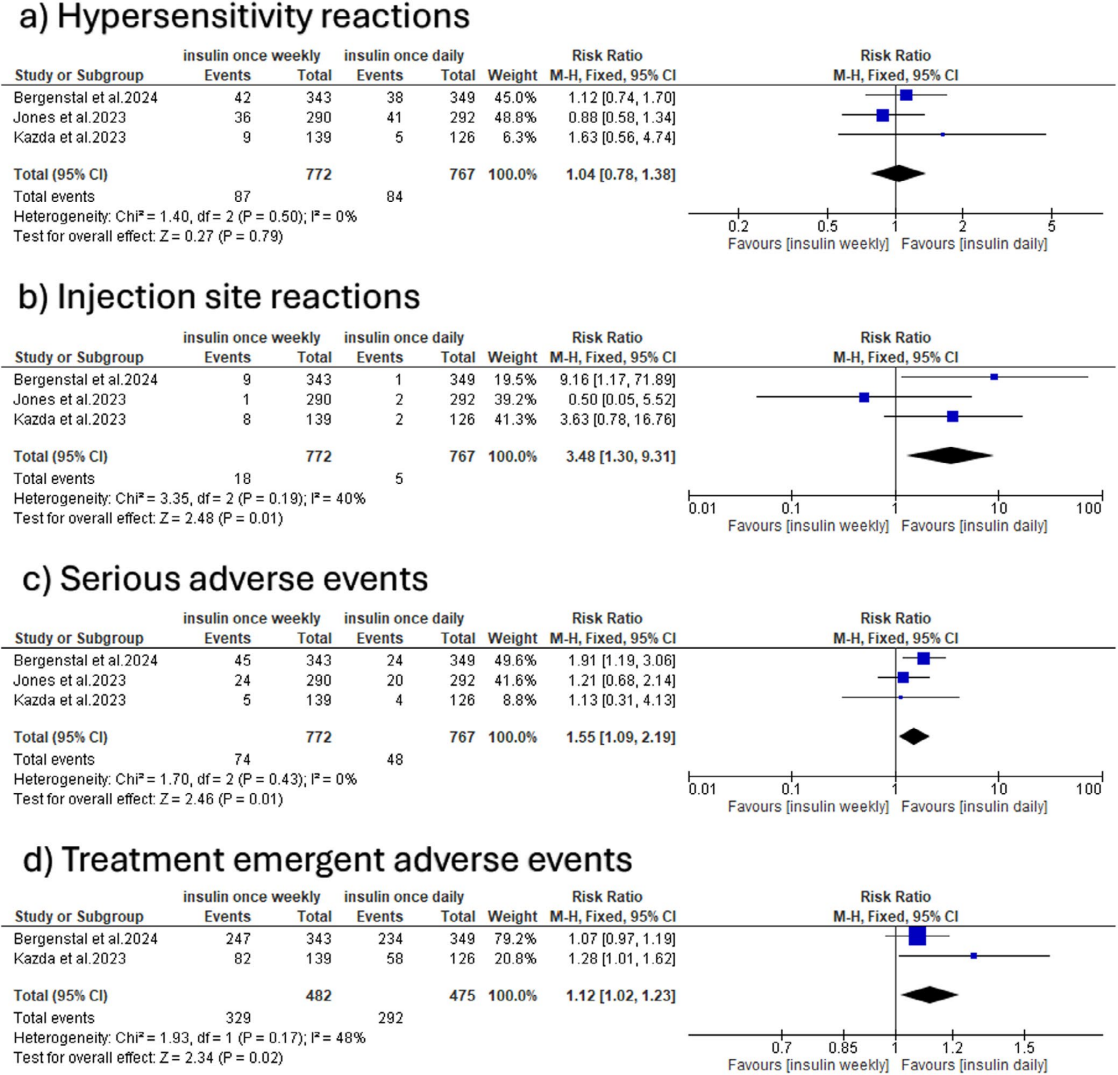
Forest plot of safety outcomes: (a) hypersensitivity reactions, (b) injection site reactions, (c) serious adverse events, and (d) treatment‐emergent adverse events.

Once‐weekly insulin was associated with a significant increase in the incidence of injection site reactions (RR: 3.48 with 95% CI [1.30, 9.31], *p* = 0.01), serious adverse events (RR: 1.55 with 95% CI [1.09, 2.19], *p* = 0.01), and treatment‐emergent adverse events (RR: 1.12 with 95% CI [1.02, 1.23], *p* = 0.02). The pooled studies were homogeneous (*p* = 0.19, *I* ^2^ = 40%), (*p* = 0.43, *I* ^2^ = 0%), and (*p* = 0.17, *I* ^2^ = 48%), respectively. However, there was no statistically significant difference between the two groups in the incidence of hypersensitivity reactions (RR: 1.04 with 95% CI [0.78, 1.38], *p* = 0.79). The pooled studies were homogeneous (*p* = 0.50, *I* ^2^ = 0%) (Figure 5a–d).

**FIGURE 6:**
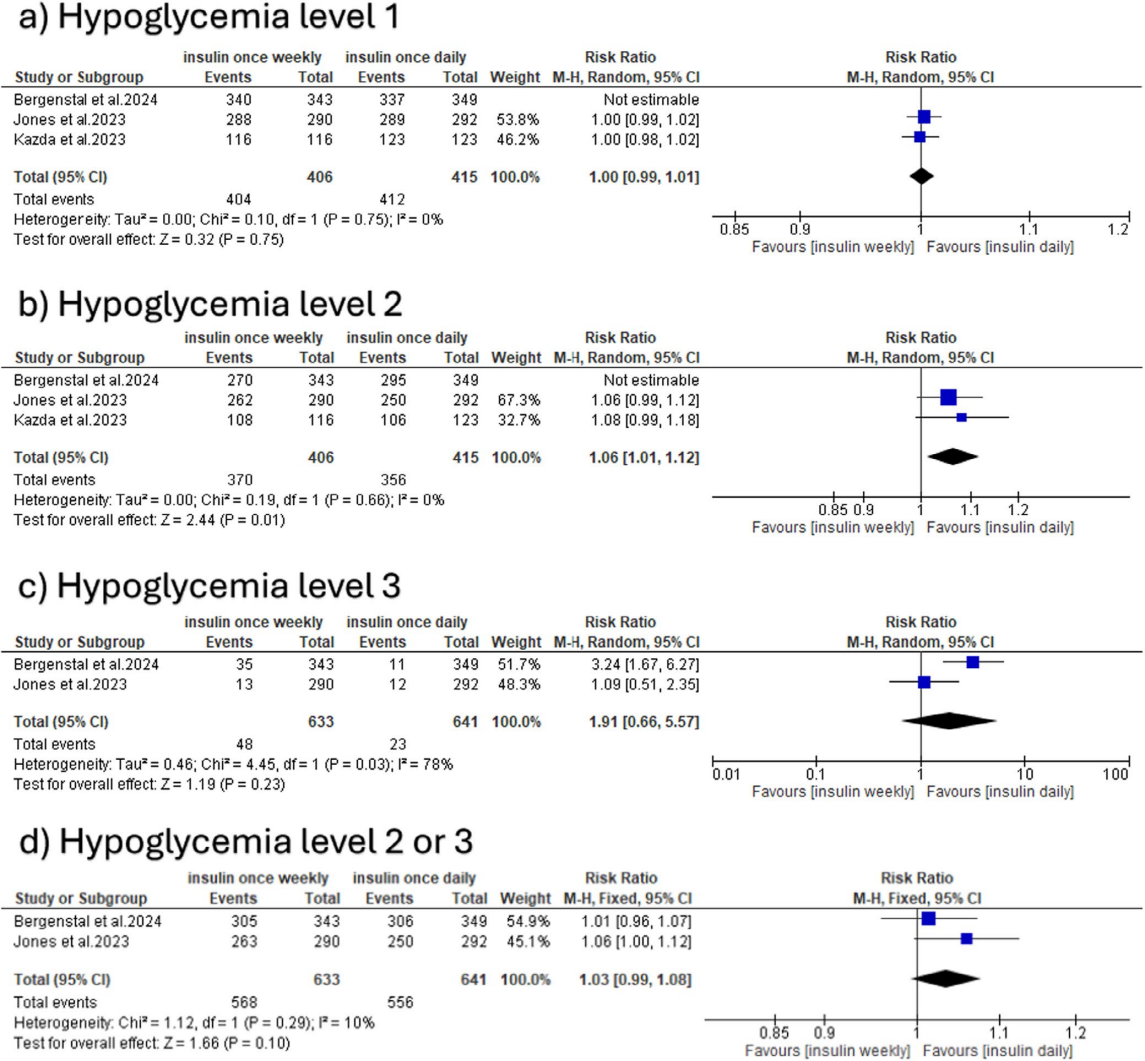
Forest plot of hypoglycemic events, categorised as (a) hypoglycemia level 1, (b) hypoglycemia level 2, (c) hypoglycemia level 3, and (d) hypoglycemia level 2 or 3.

Regarding the hypoglycemic events, there was no significant difference between the two groups in the incidence of level 1 hypoglycemia (RR: 1.01 with 95% CI [0.99, 1.03], *p* = 0.32), and the pooled studies were heterogeneous (*p* = 0.07, *I* ^2^ = 63%) (Figure 6a). However, after leaving Bergenstal et al. the pooled studies were homogeneous (*p* = 0.75, *I* ^2^ = 0%) (Table S2). Regarding the incidence of level 2 hypoglycemia, there was no significant difference between both groups (RR: 1.02 with 95% CI [0.93, 1.12], *p* = 0.69), and the pooled studies were heterogeneous (*p* = 0.008, *I* ^2^ = 80%) (Figure 6b). However, after excluding Bergenstal et al. the pooled studies were homogeneous (*p* = 0.66, *I* ^2^ = 0%), and once‐weekly insulin was associated with a statistically significant increase in the rates of level 2 hypoglycemia (RR: 1.06 with 95% CI [1.01, 1.12], *p* = 0.01) (Table S2). Meanwhile, there was no significant difference between both groups in the incidence of level 3 hypoglycemia (RR: 1.91 with 95% CI [0.66, 5.57], *p* = 0.23), and the pooled studies were heterogeneous (*p* = 0.03, *I* ^2^ = 78%) (Figure 6c). Regarding the incidence of level 2 or 3 hypoglycemia, there was no significant difference between the groups (RR: 1.03 with 95% CI [0.99, 1.08], *p* = 0.10), and the pooled studies were homogeneous (*p* = 0.29, *I* ^2^ = 10%) (Figure 6d).

**FIGURE 7:**
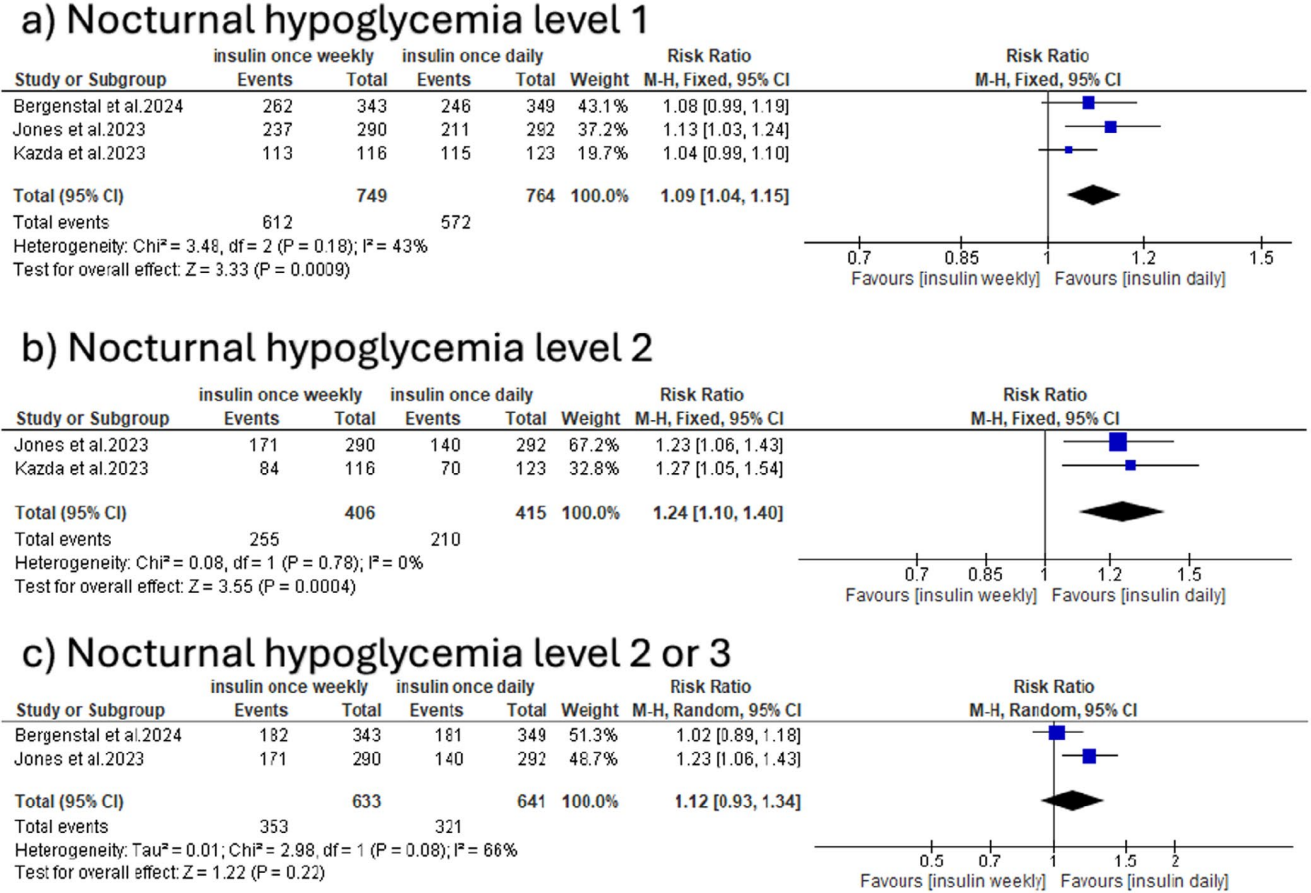
Forest plot of nocturnal hypoglycemia events, categorised as (a) nocturnal hypoglycemia level 1, (b) nocturnal hypoglycemia level 2, and (c) nocturnal hypoglycemia level 2 or 3.

Once‐weekly insulin was associated with a statistically significant increase in the incidence of level 1 nocturnal hypoglycemia (RR: 1.09 with 95% CI [1.04, 1.15], *p* = 0.0009) and level 2 nocturnal hypoglycemia (RR: 1.24 with 95% CI [1.10, 1.40], *p* = 0.0004). The pooled studies were homogeneous (*p* = 0.18, *I* ^2^ = 43%) and (*p* = 0.78, *I* ^2^ = 0%), respectively. Meanwhile, there was no significant difference in the incidence of type 2 or 3 nocturnal hypoglycemia (RR: 1.12 with 95% CI [0.93, 1.34], *p* = 0.22), and the pooled studies were heterogeneous (*p* = 0.08, *I* ^2^ = 66%) (Figure 7a–c).

## 4 Discussion

The systematic review and meta‐analysis of three RCTs, comprising 1724 participants, revealed mixed outcomes when comparing once‐weekly insulin with once‐daily insulin regimens. While once‐daily insulin demonstrated superior glycemic control with a significantly greater reduction in HbA1c, there was no significant difference in fasting blood glucose levels between the two regimens. The mean age of participants ranged from 44.1 to 47.4 years, with BMI ranging from 25.9 to 27.5 kg/m^2^.

Regarding safety outcomes, once‐weekly insulin was associated with significantly higher rates of injection site reactions, serious adverse events, and treatment‐emergent adverse events. Additionally, once‐weekly insulin showed increased rates of level 1 and level 2 nocturnal hypoglycemia. While there were no significant differences in overall level 1 and level 3 hypoglycemic events between the groups, a subgroup analysis excluding the Bergenstal study revealed that once‐weekly insulin was associated with higher rates of level 2 hypoglycemia.

Notably, between 2016 and 2018, just 21% of adults with T1D achieved the 7% HbA1c target set by the American Diabetes Association [16]. HbA1c has improved because of advancements in diabetes treatment, such as CGM [2, 6]. The primary outcome of this meta‐analysis highlights the difference in HbA1c levels. The pooled analysis of three studies, encompassing 1457 participants, revealed that once‐daily insulin was superior in reducing HbA1c compared to once‐weekly insulin. Notably, this finding was highly homogeneous across studies, suggesting consistent results across different trial settings and populations. While the difference in HbA1c is statistically significant, the small magnitude of the difference raises important questions about its clinical significance in real‐world practice. This modest difference should be carefully weighed against other factors, such as patient preference, adherence, and the practical advantages of weekly versus daily administration when making treatment decisions.

However, it is important to recognise that HbA1c reflects the average glycemic state over the past 2–3 months [17] and can be influenced by factors other than blood glucose, such as anaemia or renal impairment. Clinicians should thus account for potential confounding factors when interpreting HbA1c values and considering adjustments in insulin therapy [18].

Despite the challenges of transitioning a patient with T1DM to once‐weekly basal insulin [19], in terms of fasting blood glucose, no significant differences were observed between the two regimens. This suggests that once‐weekly insulin can achieve similar short‐term glycemic targets as daily insulin in controlling fasting blood glucose levels. However, the high heterogeneity in this outcome indicates considerable variability in the included studies, which limits the confidence in this result. Further research with standardised study designs may be needed to elucidate these regimens' impact on fasting glucose.

Despite advancements in insulin therapy, managing T1DM remains complex, and challenges persist, particularly regarding adverse reactions associated with different dosing regimens [20]. Injection site reactions, although generally rare, can range in severity from localised erythema and swelling to more generalised reactions, such as urticaria and angioedema [21]. This meta‐analysis emphasised the safety profile of once‐weekly insulin, as the less frequent dosing could potentially alter adverse event patterns. Our analysis revealed a statistically significant increase in injection site reactions among patients on once‐weekly insulin. This heightened risk may stem from the larger volume or higher insulin concentration in once‐weekly formulations, which could cause localised irritation. Further research may be necessary to optimise these formulations or adjust injection techniques to mitigate these reactions. Additionally, serious adverse events and treatment‐emergent adverse events were more frequently observed in patients using once‐weekly insulin. These findings suggest that while once‐weekly insulin offers the advantage of fewer injections, it may pose increased risks of adverse events that should be carefully considered.

Achieving optimal glycemic control with minimal glucose variability and hypoglycemia, especially nocturnal hypoglycemia, remains a key objective in insulin therapy, particularly for patients with T1DM [22]. Nocturnal hypoglycemia, defined as a drop in blood glucose levels below 3.5 mmol/L during sleep, poses a serious risk due to its potential to go undetected, leading to unrecognised hypoglycemia and an elevated risk of cardiovascular events [23]. Our analysis showed no overall significant difference in the incidence of level 1 or level 2 hypoglycemia between once‐weekly and once‐daily insulin regimens. However, excluding the study by Bergenstal et al. revealed a slight but statistically significant increase in level 2 hypoglycemia among patients on once‐weekly insulin, suggesting that this regimen may be associated with a higher risk of more severe hypoglycemic episodes. Moreover, a significant increase in nocturnal hypoglycemia was observed for both level 1 and level 2 events with once‐weekly insulin. This is particularly concerning as nocturnal hypoglycemia episodes may go undetected, increasing the risk of serious adverse outcomes. For patients with a history of hypoglycemia or those at higher risk of nocturnal episodes, clinicians should exercise caution when considering a once‐weekly insulin regimen. Enhanced glucose monitoring, particularly during nighttime hours, may be necessary to mitigate these risks. The slight increase in HbA1c observed with once‐weekly insulin may be attributed to differences in pharmacokinetics and pharmacodynamics. Once‐weekly insulins generally exhibit prolonged absorption and a flatter time‐action profile, which may lead to less stable glucose control compared to once‐daily basal insulins. Additionally, the increased risk of nocturnal hypoglycemia may stem from variations in insulin metabolism, with peak activity occurring during the overnight period [24]. Furthermore, the higher incidence of injection site reactions observed in our analysis could be related to the increased volume and concentration of the weekly formulation. These potential mechanisms highlight the need for further investigation into the long‐term metabolic effects and safety profile of once‐weekly insulin regimens.

Lastly, it is important to comment on the heterogeneity in this study, as we observed notable heterogeneity in outcomes, including fasting blood glucose levels (*p* < 0.00001, *I* ^2^ = 100%), incidence of level 1 hypoglycemia (*p* = 0.07, *I* ^2^ = 63%), level 2 hypoglycemia (*p* = 0.008, *I* ^2^ = 80%), level 3 hypoglycemia (*p* = 0.03, *I* ^2^ = 78%), and type 2 or 3 nocturnal hypoglycemia. Several factors may explain this heterogeneity. First, in the study by Jones et al. insulin dose adjustment was uniquely reported. Participants switched to insulin degludec intending to achieve a pre‐breakfast self‐measured blood glucose level of 4–7 mmol/L. The starting dose was calculated as seven times the pre‐trial daily basal insulin dose, with an additional 50% for those whose screening HbA1c was < 8.0% and 100% for those whose screening HbA1c was ≥ 8.0%. Second, the eligible HbA1c range varied across other investigations; for example, Kazda et al. included 5.6%–9.5%, whereas Bergenstal et al. included 7%–10%. Third, the target fasting glucose ranges (80–130, 80–100, and 80–120 mg/dL) differed among the three studies, potentially leading to more frequent insulin dose escalations and increasing the risk of hypoglycemia. Furthermore, CGM was only reported by Bergenstal et al. potentially affecting both the detection and management of hypoglycemia. Lastly, although both insulin icodec and insulin efsitora are administered once weekly, their pharmacokinetic profiles differ. Insulin icodec has an approximate half‐life of 7–8 days (168–196 h) [25] and reaches maximum concentration (Tmax) around 16 h post‐administration [26]. By contrast, insulin efsitora has a half‐life of approximately 17 days [8] and achieves Tmax around 4 days [26]. This prolonged half‐life supports a more stable insulin release and flatter pharmacokinetic profile, potentially reducing peak‐to‐trough fluctuations and maintaining consistent basal coverage. However, it underscores the need for new metrics that more accurately capture glycemic changes over these extended dosing intervals [26].

### 4.1 Clinical Practice Implications

Implementing once‐weekly insulin therapy in T1DM requires careful consideration of several key clinical practice elements. Patient selection is paramount, with ideal candidates being those who demonstrate poor adherence to daily regimens, maintain stable glucose control, or face significant barriers to daily insulin administration [27]. Conversely, patients requiring frequent dose adjustments, those with a history of severe injection site reactions, or individuals with highly variable insulin requirements may be less suitable for this approach. Treatment decisions should carefully weigh the trade‐offs between a slight increase in HbA1c, a higher risk of nocturnal hypoglycaemia, and potential adherence benefits, while also considering patient preferences and lifestyle factors. The monitoring strategy should be particularly rigorous during the initial transition period, incorporating more frequent blood glucose measurements, early assessment of injection sites, and close tracking of glycaemic patterns. Long‐term follow‐up should focus on regular evaluation of injection sites, monitoring for cumulative effects on glycaemic control, and ongoing assessment of patient satisfaction and adherence [28, 29, 30]. Risk mitigation strategies should address both injection site reactions through proper site rotation and technique education, as well as glycaemic control through optimised mealtime insulin dosing and potential CGM use. Comprehensive patient education is essential, covering the new administration schedule, injection site management, the importance of maintaining scheduled doses, and modified sick‐day protocols. Healthcare systems must consider various factors, including potential reductions in supply costs and healthcare utilisation, insurance coverage implications and resource allocation for increased initial education time and modified follow‐up schedules [31]. Future research priorities should address long‐term safety and efficacy, quality of life impact, cost‐effectiveness, optimal patient population identification and transition protocol development. Implementation should follow a structured approach, beginning with low‐risk patients and establishing clear monitoring protocols and communication channels. Success should be measured through systematic outcome monitoring, adverse event tracking, patient satisfaction assessment and adherence rate evaluation. While once‐weekly insulin presents a promising option for select T1DM patients, particularly those struggling with daily administration adherence, successful implementation requires careful patient selection, comprehensive monitoring and robust risk mitigation strategies within a framework of shared decision‐making between healthcare providers and patients.

### 4.2 Limitations

Several important limitations should be considered when interpreting the results of this meta‐analysis comparing once‐weekly and once‐daily insulin regimens in T1DM. The analysis was constrained by the inclusion of only three RCTs with 1724 participants, which limits the statistical power and generalisability of the findings. The relatively short follow‐up duration, ranging from 26 to 57 weeks, prevents a comprehensive assessment that limits our ability to assess long‐term glycaemic control, durability of adherence and safety outcomes. Additionally, heterogeneity was observed in fasting blood glucose outcomes, which may be influenced by differences in patient characteristics or insulin formulations across studies. Lastly, our analysis does not include real‐world data, which may provide a more comprehensive understanding of patient adherence and long‐term safety. The study population demographics were relatively narrow, with mean ages ranging from 44.1 to 47.4 years and BMI ranges of 25.9–27.5 kg/m^2^, potentially limiting applicability to broader patient populations, including elderly individuals, paediatric patients, and those with more extreme BMIs. Treatment protocols varied across studies, with two studies using alpha Efsitora and one using codec with Aspart, while comparator groups used either Degludec alone or Aspart with Degludec, making direct comparisons challenging. The assessment of outcomes focused primarily on glycaemic control and safety parameters, with limited evaluation of important patient‐centred outcomes such as quality of life, treatment satisfaction and adherence.

Additionally, the economic implications and healthcare utilisation patterns were not thoroughly examined. The analysis was further limited by varying definitions of adverse events and hypoglycemia levels across studies, potentially affecting the consistency of safety outcome reporting. Some subgroup analyses were restricted due to the small number of studies and participants, and specific patient populations may have been underrepresented. These limitations highlight the need for future research incorporating more extended follow‐up periods, larger and more diverse patient populations, standardised protocols and comprehensive outcome measures, including patient‐reported outcomes and cost‐effectiveness analyses. Particular attention should be paid to studying specific patient subgroups, including those with various comorbidities and different ethnic backgrounds, to better understand the generalisability of these findings. Despite these limitations, this meta‐analysis provides valuable initial insights into the comparative efficacy and safety of once‐weekly versus once‐daily insulin regimens while emphasising the importance of cautious interpretation and application of the findings in clinical practice.

## 5 Conclusion

In conclusion, this meta‐analysis suggests that once‐weekly insulin in Type 1 Diabetes Mellitus offers a nuanced alternative to daily insulin, with modest differences in glycemic control and notable safety considerations. While once‐daily insulin demonstrated slightly superior HbA1c reduction, once‐weekly insulin presents potential advantages in patient adherence, balanced against increased risks of injection site reactions and nocturnal hypoglycemia. Patient selection is crucial, with ideal candidates being those struggling with daily insulin regimens and maintaining stable glucose control.

## Author Contributions

Obieda Altobaishat conceived the idea. Obieda Altobaishat and Mohamed Abuelazm designed the research workflow. Obieda Altobaishat and AlMothana Manasrah searched the databases. Husam Abu Suilik and AlMothana Manasrah screened the retrieved records, extracted relevant data, assessed the quality of evidence, and Obieda Altobaishat resolved the conflicts. Ahmed Farid Gadelmawla performed the analysis. Obieda Altobaishat, Husam Abu Suilik, Ahmed Farid Gadelmawla, Mohamed Abuelazm, and Mohamed Abouzid wrote the final manuscript. Mohamed Abuelazm edited the manuscript. Obieda Altobaishat, Mustafa Turkmani, and Mohamed Abuelazm supervised the project. All authors have read and agreed to the final version of the manuscript.

## Ethics Statement

The authors have nothing to report.

## Consent

The authors have nothing to report.

## Conflicts of Interest

The authors declare no conflicts of interest.

## Supporting information


Appendix S1.

## Data Availability

The data is available upon reasonable request from the corresponding author.
