# Supplementary material for: Once‐Weekly Insulin Versus Once‐Daily Insulin for Type 1 Diabetes Treatment: A Systematic Review and Meta‐Analysis of Randomised Controlled Trials
Source: Endocrinol Diabetes Metab. 2025 Apr 4;8(3):e70048. doi: 10.1002/edm2.70048 (PMC11971483; doi:10.1002/edm2.70048)
Supplement: Supplementary file 1 — Appendix S1. [file EDM2-8-e70048-s001.docx]

**Table S1. Search query concluded on December 9, 2024.**

| Database | Search Terms | Search Field | Search Results |
| --- | --- | --- | --- |
| PubMed | ("Insulin Icodec" OR "Once-weekly insulin" OR "insulin efsitora alfa" OR "Once-Weekly Basal Insulin") AND ("diabetes mellitus" OR diabetes or diabetic or prediabetes or "insulin resistance" or "impaired glucose tolerance") | All Field | 108 |
| WOS | ("Insulin Icodec" OR "Once-weekly insulin" OR "insulin efsitora alfa" OR "Once-Weekly Basal Insulin") AND ("diabetes mellitus" OR diabetes or diabetic or prediabetes or "insulin resistance" or "impaired glucose tolerance") | All Field | 50 |
| SCOPUS | ("Insulin Icodec" OR "Once-weekly insulin" OR "insulin efsitora alfa" OR "Once-Weekly Basal Insulin") AND ("diabetes mellitus" OR diabetes or diabetic or prediabetes or "insulin resistance" or "impaired glucose tolerance") | Title, Abstract, Keywords | 164 |
| EMBASE | ("Insulin Icodec" OR "Once-weekly insulin" OR "insulin efsitora alfa" OR "Once-Weekly Basal Insulin") AND ("diabetes mellitus" OR diabetes or diabetic or prediabetes or "insulin resistance" or "impaired glucose tolerance") | All Field | 250 |
| Cochrane | ("Insulin Icodec" OR "Once-weekly insulin" OR "insulin efsitora alfa" OR "Once-Weekly Basal Insulin") AND ("diabetes mellitus" OR diabetes or diabetic or prediabetes or "insulin resistance" or "impaired glucose tolerance") | All Field | 141 |

**Table S2. Sensitivity analysis**

| **Outcome** | **No. of**  **participants (insulin weekly/insulin daily)** | **No. of**  **Trials** | **Quantitative data synthesis** | | | | **Heterogeneity analysis** | | |
| --- | --- | --- | --- | --- | --- | --- | --- | --- | --- |
|  |  |  | **MD/RR** | **95% CI** | **Z-value** | **p-value** | **df** | **p-value** | **I^2^ (%)** |
| **Fasting blood glucose** | | | | | | | | | |
| Omitting Kazda et al.2023 | 583/603 | 2 | 0.53 | [-0.98, 2.04] | 0.69 | 0.49 | 1 | P < 0.00001 | 100% |
| Omitting Jones et al.2023 | 416/437 | 2 | 0.01 | [-0.48, 0.50] | 0.03 | 0.98 | 1 | P < 0.00001 | 98% |
| Omitting  Bergenstal et al.2024 | 413/418 | 2 | 0.78 | [-0.24, 1.80] | 1.50 | 0.13 | 1 | P < 0.00001 | 100% |
| **Hypoglycemia level 1** | | | | | | | | | |
| Omitting Kazda et al.2023 | 633/641 | 2 | 1.01 | [0.99, 1.04] | 1.08 | 0.28 | 1 | 0.06 | 71% |
| Omitting Jones et al.2023 | 459/472 | 2 | 1.01 | [0.98, 1.05] | 0.74 | 0.46 | 1 | 0.02 | 83% |
| Omitting  Bergenstal et al.2024 | 406/415 | 2 | 1.00 | [0.99, 1.01] | 0.32 | 0.75 | 1 | 0.75 | 0% |
| **Hypoglycemia level 2** | | | | | | | | | |
| Omitting Kazda et al.2023 | 633/641 | 2 | 0.99 | [0.88, 1.13] | 0.11 | 0.91 | 1 | 0.007 | 86% |
| Omitting Jones et al.2023 | 459/472 | 2 | 1.00 | [0.86, 1.16] | 0.01 | 0.99 | 1 | 0.007 | 86% |
| Omitting  Bergenstal et al.2024 | 406/415 | 2 | 1.06 | [1.01, 1.12] | 2.44 | 0.01 | 1 | 0.66 | 0% |
